# MeJA regulates the accumulation of baicalein and other 4’-hydroxyflavones during the hollowed root development in Scutellaria baicalensis

**DOI:** 10.3389/fpls.2022.1067847

**Published:** 2023-01-06

**Authors:** Dali Geng, Mei Jiang, Hongjing Dong, Rongyu Wang, Heng Lu, Wei Liu, Lanping Guo, Luqi Huang, Wang Xiao

**Affiliations:** ^1^ Shandong Analysis and Test Center, Qilu University of Technology (Shandong Academy of Sciences), Jinan, China; ^2^ School of Pharmaceutical Sciences, Qilu University of Technology (Shandong Academy of Sciences), Jinan, China; ^3^ State Key Laboratory Breeding Base of Dao-di Herbs, National Resource Center for Chinese Materia Medica, China Academy of Chinese Medical Sciences, Beijing, China

**Keywords:** *Scutellaria baicalensis*, 4’-hydroxyflavone biosynthesis, root development, baicalein, hollowed root, MeJA (methyl jasmonate)

## Abstract

The dried roots of *Scutellaria baicalensis* are important traditional Chinese medicine used to treat liver and lung inflammation. An anomalous structure, hollowed root, was discovered in perennial cultivated *Scutellaria baicalensis*. The presence of the hollow may change the contents of bioactive metabolites, such as baicalein, and other 4’-hydroxyflavones in *Scutellaria baicalensis* roots, but the relationship between the hollowed root and bioactive metabolite contents is poorly understood. In this study, we identified the anatomical structure of the hollowed root and detected differentially accumulating flavonoid metabolites and enzymes related to 4’-hydroxyflavone biosynthesis in 3-year-old roots with a hollow. We confirmed that methyl jasmonate (MeJA) induced the accumulation of 4’-hydroxyflavones and the expression of enzymes related to 4’-hydroxyflavone biosynthesis in hydroponically cultured *Scutellaria baicalensis* roots. The development of the hollowed root were divided into 4 stages. The 4’-hydroxyflavone contents and expression of enzymes related to 4’-hydroxyflavone biosynthesis increased synchronously with the content of MeJA during the development of hollowed root. Pathogen and programed-cell-death related genes were induced during hollowed root development. Taken together, our results provide novel insight into the importance of MeJA in the development of hollowed root and the accumulation of 4’-hydroxyflavones in *Scutellaria baicalensis* roots. Our results suggest that a pathogen and senescence are the two major causes for the development of hollowed root in *Scutellaria baicalensis* roots.

## Introduction

*Scutellaria baicalensis* Georgi is commonly used as a traditional Chinese herb, known as Huang-Qin. Huang-Qin has more than a 2,500-year history in the treatment of fever, and shows anti-viral and anti-inflammatory activities against the severe acute respiratory syndrome coronavirus 2019 (Liu et al., 2021; Song et al., 2021). Huang-Qin has been reported to be curative in combination therapies for non-small cell lung carcinoma (Duan et al., 2014).

The major medicinal metabolites in *S. baicalensis* are 4’-hydroxyflavones, including baicalein, wogonin, and scutellarein. Together with their glucosides baicalin, wogonoside, and scutellarin, 4’-hydroxyflavones provide antiviral, anticancer, and anti-inflammatory activities (Himeji et al., 2007; Nayak et al., 2014; Wang et al., 2018). The National Pharmacopoeia determined that baicalin content is the main evaluation index and quality control of *S. baicalensis* (National Pharmacopoeia Committee, 2015).

**Figure 1:**
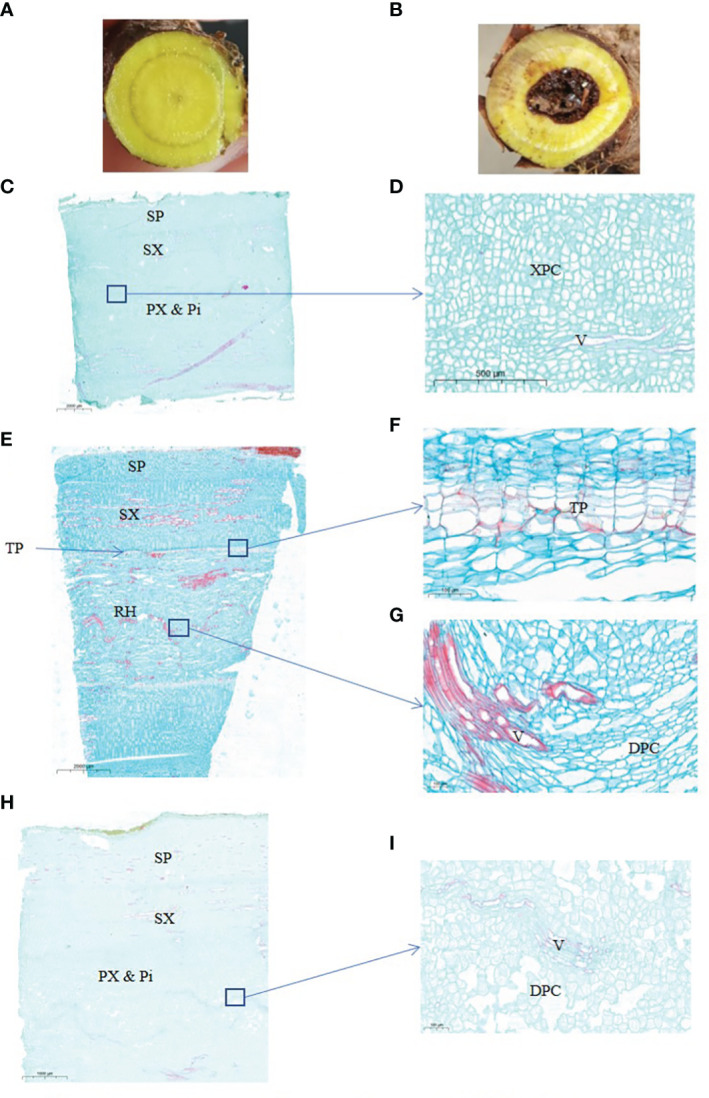
Anatomical structure of 2-years and 3-years root of S. baicalensis. **(A)**, cross section of 2 years roots. **(B)**, cross section of 3 years roots. **(C)**, vertical section of 2 years roots. **(D)**, zoom in on the central part of **(C, E)**, vertical section of 3 years roots with a hollow. **(F)**, zoom in on the ternary phellem tissue of **(E, G)** zoom in on the hollowed root of **(E, H)**, vertical section of 3 years roots without a hollow. **(I)** Zoom in on the central part of **(H)** SP, secondary phloem. SX, secondary xylem. PX & Pi, primary xylem and pith. XPC, xylem parenchyma cells. V, vessels. TP, ternary phellem. DPC, dead parenchyma cells.

Biosynthesis of 4’-hydroxyflavones in *S. baicalensis* roots has been described (Zhao et al., 2016; Zhao et al., 2018; Zhao et al., 2019), but the regulation of 4’-hydroxyflavone biosynthesis and its accumulation remain unknown. The accumulation of 4’-hydroxyflavones is related to a unique anatomical structure called the “hollowed root” (**Figure 1B**). The content of 4’-hydroxyflavone glucosides decreases in roots with a hollow, but the content of 4’-hydroxyflavones increases compared with roots without a hollow (Ren et al., 2009; Zhao et al., 2018). Although roots with a hollow have been recorded in classical prescriptions and medical classics, the theoretical basis for the development of hollowed root, and the relationship between the hollowed root and 4’-hydroxyflavone contents remains unclear.

Jasmonic acids (JAs), including jasmonic acid and its oxylipin derivatives like Methyl jasmonate (MeJA) are hormones that regulate plant senescence and the defense response (Ren et al., 2010; Wasternack and Hause, 2013; Zhou and Memelink, 2016). JAs induce the production of 4’-hydroxyflavones in cultured *S. baicalensis* cells (Xu et al., 2010), and JAs are induced when a plant is attacked by a pathogen (Cheong and Choi, 2003) or enters senescence (Ghorbel et al., 2021). Exogenous MeJA induce the accumulation of phenolic phytoalexins, such as flavonoids in *Arabidopsis thaliana, Vitis vinifera, Malus domestica*, and *Musa acuminata* (Shafiq et al., 2011; De Geyter et al., 2012; Portu et al., 2015). JAs also promote leaf senescence in *A. thaliana* (Pandey et al., 2016). Endogenous JA levels are four-fold higher in senescent leaves than those in non-senescent leaves (Qi et al., 2015). Genes associated with the JA biosynthetic pathway are induced during leaf senescence (He et al., 2002; Schommer et al., 2008; Qi et al., 2015).

The multiple roles of JAs in plant senescence and the defense response inspired our hypothesis on the relationship between hollowed root and 4’-hydroxyflavone contents. In this study, we revealed that the accumulation of 4’-hydroxyflavones during development of the hollowed root were regulated by MeJA, and the accumulation of 4’-hydroxyflavones was related to senescence of xylem cells and biotic stress.

## Materials and methods

### Plant materials

The plants were maintained at Laiwu, Shandong Province, China (36°20´N, 117°47´E). S. baicalensis Georgi was planted in pots (22 × 60 cm) filled with equal parts of local loess soil and sand. The pots were buried in the soil in an outdoor garden. The S. baicalensis seeds were planted in the pots in March each year.

The 2- and 3-year-old plants were labeled in March, and 20 of the 2- and 3-year- old plants were carefully removed from the soil and washed. The clean roots were cut at root-shoot vascular transition zone. Then, 3-4 cm root segments were cut out as shown in **Supplementary Figure 1**. The root segments were vertically sectioned at a one-third circle, and the root segments were divided into two parts. The larger part was vacuumed packed in FAA stationary solution (5% [v/v] formalin, 5% [v/v] acetic acid, and 90% [v/v] ethyl alcohol) for 30 min and then fixed for 7 days. The smaller part was snap frozen in liquid nitrogen and stored at −80°C. The larger part was prepared for a morphological analysis, and the smaller part was prepared for metabolomics, transcriptomics, and the qRT-PCR analysis.

Twelve of the 2-year-old plants were harvested after March, and prepared as described above every 2 weeks until October. Photographs of cross-sections of these roots segments were taken with a digital camera (D80, Nikko, Tokyo, Japan) before the vertical sections were prepared. These samples were marked as rotten heart developing samples with the harvest date.

The 3-month old plants were carefully removed from the soil and washed for the MeJA, SA and ABA treatment. They were transferred into plastic containers containing 7 L of Hoagland solution for an additional 1 month. All plants were hydroponically cultured in a growth chamber at 25°C, with illumination of 60 μmol m^-2^ s^-1^, and humidity of 50-75%. Then plants were transfered into hydroponic solution with 700 µM MeJA, 500µM SA,10µM ABA (Sigma, St. Louis, MO, USA) or control, respectively, for 6, and 72 h. The treatment solution were changed everyday to maintain concentration of exogenous hormones stable. At the end of each treatment, the roots of control and treatment groups were washed and two 1-cm long root segments were cut; one was snap frozen in liquid nitrogen and the other was fixed in FAA stationary solution. The samples were stored at −80°C. The 6 h samples were used for qRT-PCR analysis, the 72 h samples were used for the UPLC-MS analysis, and the 0 h samples were the control. Root morphological analysis and the hollowed root developmental stages.

The fixed roots were transferred to 10% (v/v) ethylenediamine at 55°C for 5 days of dissociation. The roots were subsequently dehydrated through a graded ethanol series for 4 h (30%, 50%, 75%, 85%, 95%, and 100% twice, v/v). Transparent roots obtained from the sequential xylene treatment were embedded in paraffin. The embedded blocks were sectioned with a rotary microtome (RM2125RTS, Leica, Jena, Germany) and dyed with saffron and fast green. The sections were observed with a light microscope (E-100, Nikko, Japan). Photographs were taken with a digital camera (DS-U3, Nikko, Japan) mounted on the microscope. Ten root segments from 2- and 3-year-old roots samples and 9 root segments of developing hollowed root samples were analyzed.

### Preparation of the sample powder

The smaller parts of the root segments that were frozen in liquid nitrogen were crushed in a mixer mill (MM 400, Retsch) containing small steel balls in liquid nitrogen for 1.5 min at 30 Hz. The powder was divided into equal parts. One part was stored at −80°C, and the other part was freeze-dried in a vacuum freeze-dryer (N-10, Scientz Biotechnology, Ningbo, Zhejiang Province, China). The lyophilized powder was stored in −80°C. Three root segments were used as biological repeats.

### Metabolomics analysis

Lyophilized powder of the 2- and 3-year-old roots with a hollow was used for the metabolomics analysis. The metabolomics analysis was performed by Metware Biotechnology (Wuhan, Hubei Province, China) with methods described by Chen et al. (2013). Each group (2- and 3-year-old roots with hollows) had 3 biological repeats.

### qRT-PCR analysis

The unlyophilized powder of the hollowed root samples was used for the qRT-PCR analysis. A 500 mg portion of unlyophilized powder was used to extract RNA. The RNA was extracted with the RNAprep Pure Plant Kit (#DP441, Polysaccharides & Polyphenolics-rich, Tiangen Biotech, Beijing, China). Two µg of total RNA was used to synthesize the first-strand cDNA with the PrimeScript™ 1st Strand cDNA Synthesis Kit (Takara, Shiga, Japan). The cDNA reaction mixture was diluted five times, and 5 µl was used in the 20-µl PCR reaction. The PCR reactions included a pre-incubation step at 95°C for 2 min followed by 45 cycles of denaturation at 95°C for 15 s, annealing at 54°C for 30 s, and extension at 72°C for 30 s. All reactions were performed in the QuantStudio™ 5 Food Safety Real-Time PCR System using TB Green Fast qPCR Mix (Takara) and ROX reference dye. Each experiment had 9 replicates (3 technical replicates for each biological replicate). The primers are listed in **Supplementary Table 3**. The relative expression level were calculated by Delta-delta Ct values.

### UPLC-MS analysis

Baicalin, baicalein, wogonin, wogonoside, pinocembrin, pinocembrin chalcone, chrysin, and MeJA were purchased from Desite Biotech (Chengdu, Sichuan Province, China). Standard stock solutions (0.1 mg ml^-1^) were prepared by dissolving the compounds in methanol. Working standard solutions were obtained by serially diluting the stock solutions with methanol.

Lyophilized powder was used for the UPLC-MS analysis. A 100 mg sample was extracted with 1.5 mL of 50% methanol in a sonicator bath for 1 h, and centrifuged at 12,000 × g for 5 min to remove debris. The supernatant was filtered through a 0.2-μm filter before injection. UPLC was performed with the Acquity H (Waters Corp., Milford, MA, USA). MS was performed with the Xevo TQ-XS system (Waters Corp). Separation was achieved with a 150 × 2.1 mm 3 µm C18-120 column (Shimadzu, Tokyo, Japan) and the following gradient: 0.1% formic acid in water (A) vs. 0.1% formic acid in acetonitrile (B) run at 0.3 mL min^−1^ and a column temperature of 40°C (0 min, 95% B, 15 min, 95% B, 16 min, 95% B, 17 min, 5% B, 20 min, 5% B). MS detection followed the method: baicalin, m/z 445, 269; baicalein, m/z 269, 120.8; wogonin, m/z 283, 268; wogonoside, m/z 459, 283; pinocembrin, m/z 255, 213; chrysin, m/z 255, 153; pinocembrin chalcone, m/z 257,239,215. MeJA, m/z 225, 150.9. All metabolites were scanned in negative ESI mode, except pinocembrin chalcone and MeJA; these two metabolites were scanned under positive ESI mode. The spray chamber conditions were 50 U of sheath gas, 5 U of auxiliary gas, 300°C capillary temperature, and 3.8-kV spray voltage. Each experiment had 5 biological replicates.

### RNA-seq analysis

Lyophilized powder from 2- and 3-year-old roots with a hollow was used for the RNA-seq analysis. The RNA-seq analysis was performed by Metware Biotechnology (Wuhan, Hubei Province, China) with the method described by Wang et al. (2009). Each group (2- and 3-year-old roots with a hollow) had 3 biological repeats.

### Gene clone and phylogenetic analysis

The cDNA reaction mixture was used for cloning. Gene cloning were performed with TaKaRa Ex Taq^®^ (Takara, Japan). The PCR products were subcloned into pDONER 207 using the Gateway BP Clonase II enzyme mix (Thermo Fisher). Sequenced genes were aligned and the phylogenetic trees were built using Molecular Evolutionary Genetics Analysis version 7.0 (Kumar et al., 2016), maximum likelihood methods with 1000 replicate bootstrap support.

### Statistics

All data are presented as mean ± SD. Paired or unpaired two-tailed Student’s t- tests were used to compare group differences. P-values < 0.05 were considered significant. For all UPLC-MS analysis, 5 biological repeats were used. For all qRT-PCR, transcriptome and metabonomic analysis, 3 biological repeats were used.

## Results

### Anatomical structure of the *S. baicalensis* hollowed root

The hollowed root of *S. baicalensis* develops with age. To investigate the changes during the development of the hollowed root of *S. baicalensis*, we chose 2- and 3-year-old roots for a paraffin section analysis. The appearance of the *S. baicalensis* roots from different years is shown in **Figures 1A, B**. Hollow was discovered in 3-year-old roots but not in 2-year-old roots. Not all 3-year-old roots developed hollow. Vertical sections of the hollowed root from 2- and 3-year-old plants are shown in **Figures 1C–I**. The vertical sections show that the hollow consists of dead secondary xylem surrounded by ternary phellem tissue (**Figures 1F–H**), indicating that hollow is dead xylem tissue. Dead parenchyma cells arranged in rows were discovered in a vertical section of the xylem in some 3-year-old roots without a hollow, but no ternary phellem tissue was seen (**Figures 1I, J**).

### Metabolomic analysis of *S. baicalensis* roots reveals changes in flavonoids in different ages of roots

**Figure 2:**
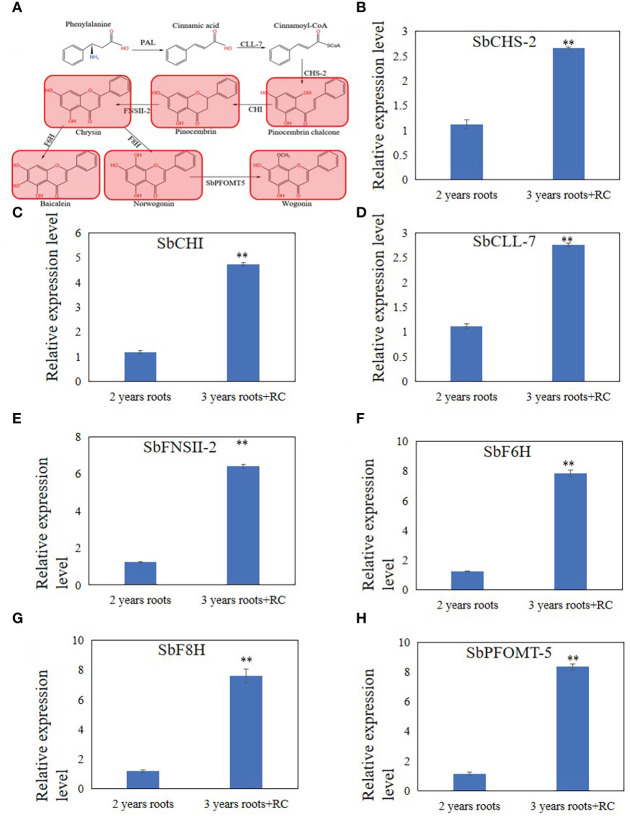
Differential expression metabolites and genes related to 4'deoxyflavones biosynthesis discovered by metabolome and qRT-PCR analysis in 3 years roots with a hollow. **(A)**, Differential expression metabolites discovered by metabolome analysis. Read color indicate metabolites up-regulated in 3 years roots with a hollow. **(B-H)**, relative expression level of SbCHS-2 **(B)**, SbCHI **(C)**, SbCLL-7 **(D)**, SbFNSII-2 **(E)**, SbF6H **(F)**, SbF8H **(G)** and SbPFOMT-5 **(H)**. The relative expression level were calculated by Delta-delta Ct values. The data are the means ± SDs (n = 3), **means *p* < 0.01.

To understand the changes in flavonoid metabolites during senescence of *S. baicalensis* roots, targeted liquid chromatography-tandem mass spectrometry-based metabolomics was applied to the 2- and 3-year-old roots with a hollow. Principal component analysis (PCA) of the metabolomic profiles is shown in **Supplementary Figure 2A**. The results indicate that the flavonoid metabolites presented distinct variations between 2- and 3-year-old roots with a hollow (**Supplementary Figure 2A**). All metabolites detected are shown in **Supplementary Table 1**. The orthogonal projections to latent structures discriminant analysis indicated that the Variable Importance in Projection (VIP) value had satisfactory predictive capabilities (**Supplementary Figure 2B**). The differentially expressed metabolites (DEMs) were filtered by VIP > 1. Among all flavonoid metabolites, 95 DEMs were filtered in 2-year-old roots vs. 3-year-old roots with a hollow, and the clustered heatmap is shown in **Supplementary Figure 2C**. Forty-three of the 95 DEMs were flavonoids and 52 were flavonoid glycosides. The contents of 9 flavonoid and 8 flavonoid glycosides DEMs were lower in 3-year-old roots with a hollow than those in 2-year-old roots (**Supplementary Table 1**). 36 others flavonoid and 43 other flavonoid glycosides DEMs were higher in 3-year-old roots with a hollow than those in 2-year-old roots (**Supplementary Table 1**). KEGG analysis was performed to confirm the location of the flavonoid DEMs in the flavonoid biosynthetic pathway (**Supplementary Figure 3**). Most of the KEGG annotated DEMs were enriched in the flavonoid biosynthetic pathway (**Supplementary Figure 3A**) and fewer were enriched in the flavone and flavonol biosynthetic pathways (**Supplementary Figure 3B**). Differences were detected in the baicalein biosynthetic pathway. (**Figure 2A**). The chrysin, pinocembrin, pinostrobin chalcone, baicalein, norwogonin, and wogonin contents were higher in 3-year-old roots with a hollow than those in 2-year-old roots. The contents of baicalin, scutellarin, and wogonoside were higher in 2-year-old roots than those in 3-year-old roots with a hollow.

### Real-time quantitative polymerase chain reaction (RT-qPCR) analysis revealed expression changes in baicalein biosynthetic enzymes in *S. baicalensis* roots

To explain the differences in the accumulation of baicalein and other flavonoids in 2- and 3-year-old roots with a hollow, RT-qPCR analysis of SbCLL-7, SbCHS-2, SbCHI, SbFNSII-2, SbF6H, SbF8H, SbPFOMT-5 was performed to examine the expression levels of enzymes related to baicalein biosynthesis that were reported by Zhao et al. (2016; 2018; 2019). The results showed that the relative expression levels of SbFNSII-2, SbCHI, SbCLL-7, SbF6H, SbF8H, and SbPFOMT-5 increased in 3-year-old roots with a hollow compared with 2-year-old roots (**Figures 2B–H**).

### MeJA regulate accumulation of flavonoids during developing of hollowed root

**Figure 3:**
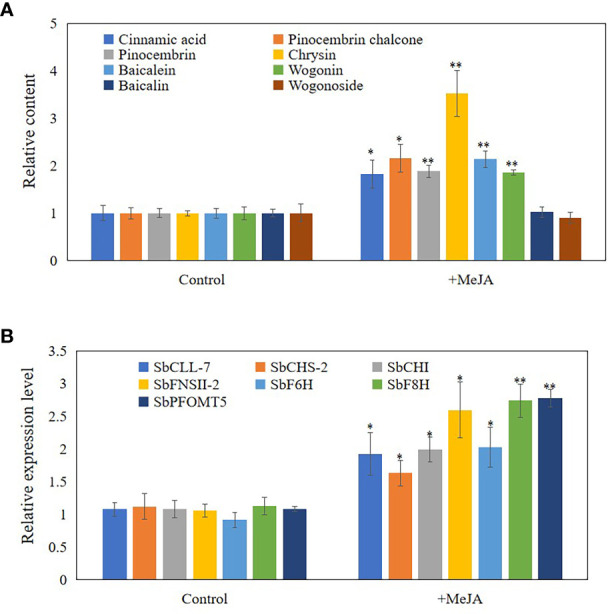
Content and expression level of metabolites and enzymes related to 4'-deoxyflavones biosynthesis in hydroponic Scutellaria baicalensis roots with treatment of MeJA. **(A)**, relative content of metabolites related to 4'-deoxyflavones biosynthesis. The data are the means ± SDs (n = 5), *means means p < 0.05, **means p < 0.01. **(B)**, relative expression level of enzymes related to 4'-deoxyflavones biosynthesis. The data are the means ± SDs (n = 3), *means means p < 0.05, **means p < 0.01.

As JAs regulate the accumulation of flavonoids (Xu et al., 2010), senescence (Ghorbel et al., 2021), and programmed cell death (PCD) (Reinbothe et al., 2009), we hypothesized that JAs may induce the development of hollowed root and the accumulation of 4’-deoxyflavones in 3-year-old roots with a hollow. Exogenous MeJA was applied to hydroponic roots of *S. baicalensis* to confirm the role of JAs in the accumulation of 4’-deoxyflavones and the development of hollowed root. The 4’-deoxyflavone contents and the expression of enzymes related to baicalein biosynthesis were induced by MeJA (**Figure 3**), but no hollowed root developed (**Supplementary Figure 4**). Content and expression level of metabolites and enzymes related to 4’-deoxyflavones biosynthesis were not response to abscisic acid (ABA) and salicylic acid (SA) treatment (**Supplementary Figure 5**).

**Figure 4:**
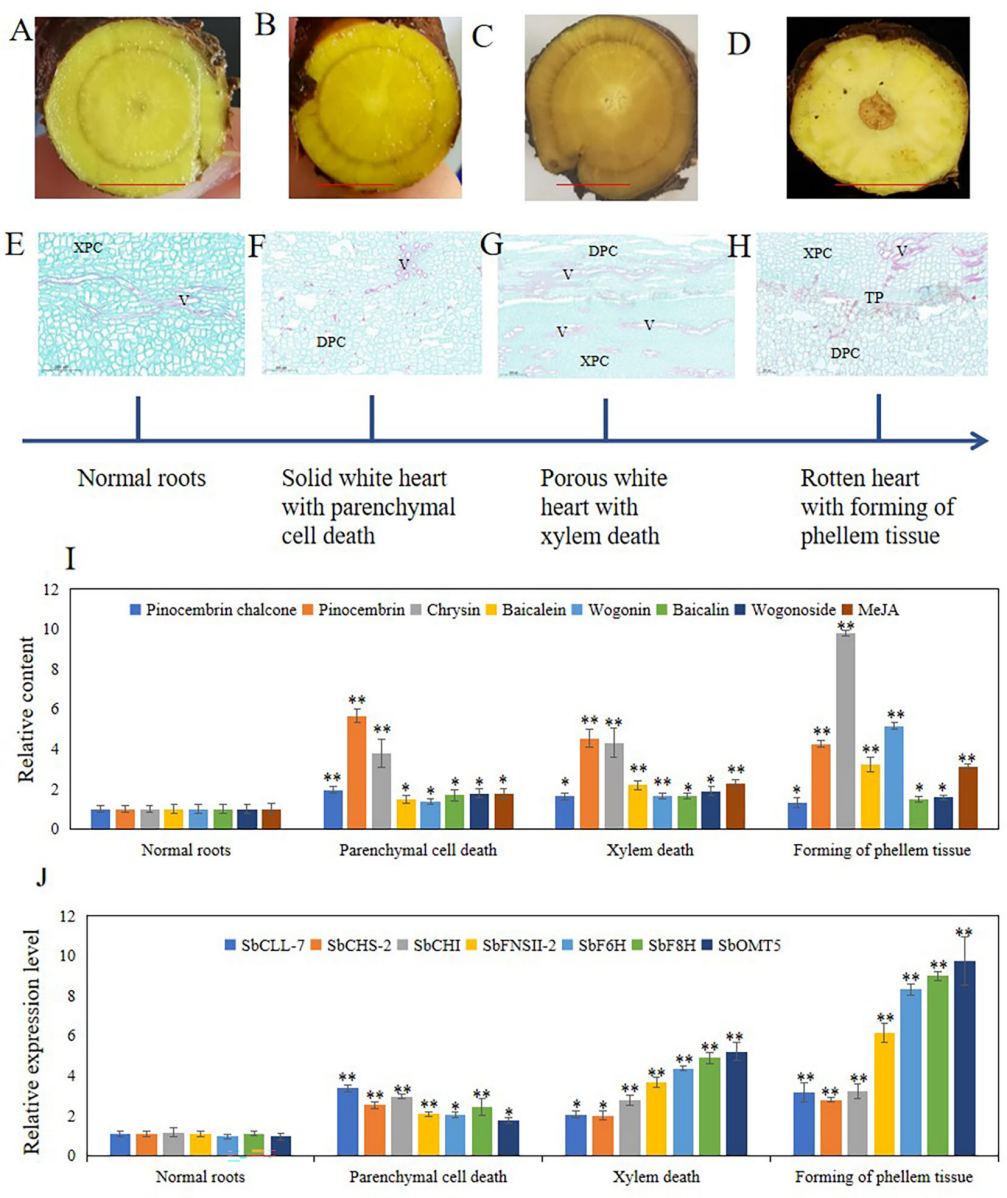
Four stages of hollowed roots development and relative content of metabolites related to 4’-deoxyflavones biosynthesis and relative expression level of genes related to 4’-deoxyflavones biosynthesis in each stage. **(A-D)**, cross section of roots in 4 stages, bar represent 1 cm. **(E-H)**, vertical section of roots in 4 stages. **(I)**, relative content of metabolites related to 4’-deoxyflavones biosynthesis, The data are the means ± SDs (n = 5). **(J)**, relative expression level of genes related to 4'-deoxyflavones biosynthesis, The data are the means ± SDs (n = 3). *means *p* < 0.05, **means *p* < 0.01. XPC, xylem parenchyma cells. V, vessels. TP, ternary phellem. DPC, dead parenchyma cells.

Hollowed root consists of dead xylem tissue surrounded by ternary phellem tissue, and the development of phellem tissue takes a long time. To clarify the development stage of hollowed root in *S. baicalensis* roots, we continuously collected roots from *S. baicalensis* for the first 6 months after 2-years, and vertical sections were prepared from these roots. The development of hollowed root was divided into 4 stages based on the anatomical structure: 1) normal roots, no changes. 2) Solid white heart with death of parenchymal cells, which have a solid white heart. Dead parenchymal cells were observed in the pith and primary xylem. 3) Porous white heart with dead xylem. Dead primary xylem is observed. 4) hollowed root with the formation of phellem tissue. Ternary phellem tissue was observed (**Figures 4A–H**). The 147 hollowed root samples were divided into these 4 stages: 52 samples were stage 1, 27 samples were stage 2, 18 samples were stage 3, and 41 samples were stage 4. Nine samples were excluded because the paraffin sections were poor.

To clarify the role of JAs in the development of hollowed root and the accumulation of 4’-deoxyflavones, 4’-deoxyflavone and MeJA contents and the expression of enzymes related to 4’-deoxyflavone biosynthesis were analyzed by ultra-performance liquid chromatography-mass spectrometry (UPLC-MS) and qRT-PCR in the 4 developmental root stages (**Figure 4I**). MeJA and 4’-deoxyflavone contents increased synchronously during the development of hollowed root. A significant increase in stage 4 roots was observed compared with stage 3 roots. The expression of enzymes related to 4’-deoxyflavone biosynthesis increased synchronously with MeJA. Significant increases were also observed in stage 4 compared with stage 3 roots (**Figure 4J**).

### RNA-seq analysis discovers genes related to the accumulation of 4’-deoxyflavones, PCD and pathogen response

RNA-seq analysis was performed on 2- and 3-year-old roots with a hollow to discover the differentially expressed genes (DEGs) involved in the expression of enzymes related to the development of hollowed root and baicalein biosynthesis. PCA clearly separated the 2-year-old from the 3-year-old roots with a hollow (**Supplementary Figure 6A**), and Pearson’s correlation results indicated a perfect in-group correlation (**Supplementary Figure 6B**). We chose genes with |log2FoldChange| > 1 and a q-value < 0.05 as DEGs. As a result, 465 DEGs were detected. The expression levels of all DEGs are shown by the heatmap and volcano plots in **Supplementary Figures 6C, D**.

The 465 DEGs were annotated through eggNOG-mapper (Huerta-Cepas et al., 2019; Cantalapiedra et al., 2021) and blasted to the Swiss-Prot database (UniProt Consortium, 2021) to annotate the functions of the genes (**Supplementary Table 2**). In total, 370 of 463 DEGs had significant blast results. We discovered that 41 DEGs were annotated to the biosynthesis of flavonoids, resistance to biotic stress, PCD, and the development of xylem and phellem tissues (**Supplementary Table 2**). Among all 41 DEGs, 7 reported 4’-deoxyflavone biosynthetic enzymes were discovered, including Sb01g34300, Sb03g20740, Sb09g15450, Sb03g24340, Sb09g03510, and Sb01g39830 (Zhao et al., 2019). Sb01g34300 was SbPFOMT5, Sb03g20740 was SbFNSII-2, Sb09g15450 was SbCLL-7, Sb03g24340 was SbCHI, Sb09g03510 was SbCHS-2, and Sb01g39830 was SbF8H. The RNA-seq results of these genes were consistent with our qRT-PCR results (**Figure 4**). Sb01g50771 was reported to be baicalein 7-O-glucuronosyltransferase, which catalyzes the biosynthesis of baicalin (Nagashima et al., 2000). Sb01g50771 expression was induced in 3-year-old roots with a hollow (**Supplementary Table 2**).

Twenty-four of the 41 DEGs were annotated to resistance to pathogens and PCD. Among these 24 genes, 17 were upregulated and 7 were downregulated. Sb01g32610 was annotated as a homolog of AtMC9, and Sb01g53940 was annotated as a homolog of AtMC1. These two metacaspases control the progress of PCD in *A. thaliana*, indicating that PCD participates in the development of hollowed root (Watanabe and Lam, 2005; Bollhöner et al., 2013). Sb01g32610 and Sb01g53940 expression was induced in 3-year-old roots with a hollow (**Supplementary Table 2**). Sb01g06320 was annotated as a homolog of AtPGIP2, which is a polygalacturonase inhibitor induced by fungi (Ferrari et al., 2006). Sb01g16540 and Sb01g16570 were annotated as homologs of AtRPP8 and AtRPP13, which are plant disease resistance genes (R genes) that respond to fungal infection. The expression of Sb01g06320, Sb01g16540, and Sb01g16570 was induced in 3-year-old roots with a hollow.

Ten of the 41 genes were annotated to the development of xylem and phellem tissues. Among these 10 genes, 7 were upregulated and 3 were downregulated. Sb08g10770 was annotated as a homolog of AtWOX4, a key regulator of periderm development (Xiao et al., 2020). Sb08g10770 expression was induced in 3-year-old roots with a hollow (**Supplementary Table 2**).

**Figure 5:**
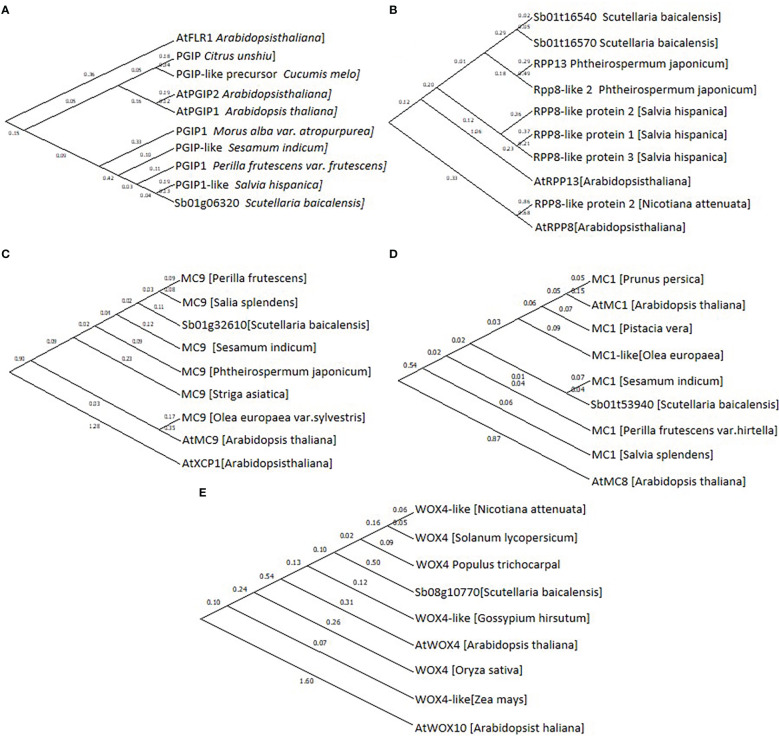
Phylogenetic tree of **(A)**, Sb01g06320; **(B)**, Sb01g16540 and Sb01g16570; **(C)**, Sb01g32610; **(D)**, Sb01g53940 and **(E)**, Sb08g10770. Maximum likelihood (ML) was used to construct this tree with 1000 replicate bootstrap support.

Based on their annotation, CDS of Sb01g32610, Sb01g53940, Sb08g10770, Sb01g06320, Sb01g16540, and Sb01g16570 were cloned and sequenced, then phylogenetic tree was performed to confirmed their annotation (**Figure 5**).

### Genes related to the response to pathogens and the development of periderm were only differentially-expressed in stage 4 roots.

**Figure 6:**
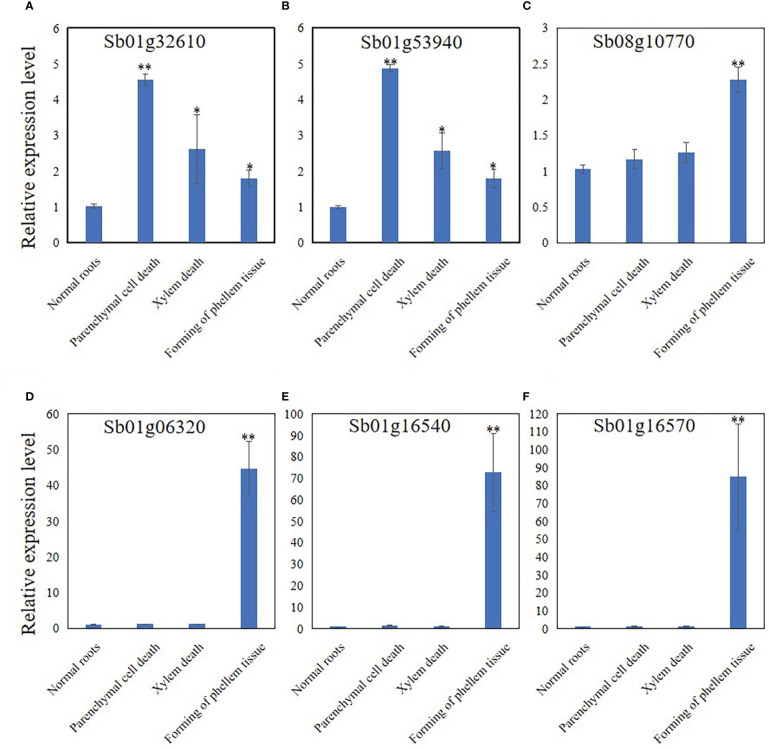
Relative expression level of Sb01g32610 **(A)**, Sb01g53940 **(B)**, Sb08g10770 **(C)**, Sb01g06320 **(D)**, Sb01g16540 **(E)** and Sb01g16570 **(F)** in root of 4 stages during hollowed roots development. The relative expression level were calculated by Delta-delta Ct values. The data are the means ± SDs (n = 3). *means *p* < 0.05, **means *p* < 0.01.

The expression levels of Sb01g32610, Sb01g53940, Sb08g10770, Sb01g06320, Sb01g16540, and Sb01g16570 were analyzed by qRT-PCR to discover the expression pattern of genes related to PCD, development of phellem tissue, and the response to pathogens. The results showed that the expression of Sb01g32610 and Sb01g53940 was significantly induced at stage 2 (**Figures 6A, B**). The expression of Sb01g32610 and Sb01g53940 decreased in stage 3 and 4 roots, compared with stage 2 roots, but was still induced compared with stage 1 (**Figures 6A, B**). The expression of Sb08g10770, Sb01g06320, Sb01g16540, and Sb01g16570 was only significantly induced during stage 4 (**Figures 6C–F**). The expression levels of Sb01g32610, Sb01g53940, Sb08g10770, Sb01g06320, Sb01g16540, and Sb01g16570 in stage 4 roots were consistent with our RNA-seq results (**Figure 6**).

## Discussion

*S. baicalensis* is noted for its high 4’-deoxyflavone and 4’-deoxyflavone glycoside contents in roots. The presence of the hollowed root in *S. baicalensis* roots, and the accumulation of 4’-deoxyflavones are related. Our research aimed on these two focus: 1) Many flavonoids, not only 4’-deoxyflavones, were induced when hollowed root is present 2) The development of hollowed root and the accumulation of 4’-deoxyflavones related to each other by JAs.

### Flavonoids in *S. baicalensis* are induced in roots with a hollow compared with roots without a hollow

The metabolomic results revealed 98 differentially expressed flavonoids and flavonoid glycosides and 78 of them were upregulated in 3-year-old roots with a hollow (**Supplementary Figure 2C**, **Supplementary Figures 3A, B**), including flavonoids related to the 4’-deoxyflavone biosynthetic pathway (**Figure 2A**) and enzymes related to 4’-deoxyflavone biosynthesis (**Figures 2B–H**). These results indicate that the accumulation of 4’-deoxyflavones is not unique, and that many other flavonoids were induced in roots with a hollow. The 4’-deoxyflavone biosynthetic pathway in *S. baicalensis* roots is a branch of flavonoid biosynthesis (Zhao et al., 2016; Zhao et al., 2018; Zhao et al., 2019). The induction of flavonoids indicates that 4’-deoxyflavones are not uniquely induced metabolites when hollowed root is present.

### Accumulation of 4’-deoxyflavones in roots is induced by JAs, and JAs may be induced by senescence in xylem cells.

Hollowed root only developed in 3-year or older roots of *S. baicalensis*, but not all 3-year-old roots contained hollowed root. This developmental model is very similar to the development of heartwood in trees, which also only develops in old secondary xylem, and flavonoids accumulate during development (Celedon and Bohlmann, 2018; Xiao et al., 2020).

JAs enhance the expression of several genes in the phenylpropanoid pathway and the products accumulate in *S. baicalensis* roots (Xu et al., 2010; Zhao et al., 2016). JAs also regulate root development (Zhu et al., 2006), xylem formation (Jang et al., 2019), defense and PCD (Reinbothe et al., 2009). Thus, we hypothesized that JAs may induce the expression of enzymes related to baicalein biosynthesis and the accumulation of 4’-deoxyflavones. Exogenously treating hydroponically cultured roots with MeJA substantiated our hypothesis (**Figure 3**). The contents of 4’-deoxyflavones and the expression of enzymes related to baicalein biosynthesis were induced by MeJA (**Figure 3**). 4’-Deoxyflavone glucoside contents were not induced or suppressed by MeJA in roots treated with MeJA (**Figure 3B**). These results indicate that the accumulation of 4’-deoxyflavone glucosides may be regulated in several ways.

Development of hollowed root was divided into 4 stages by its anatomical structure: 1) normal roots. 2) Solid white heart with dead parenchymal cells. 3) Porous white heart with dead xylem. 4) hollowed root and the formation of phellem tissue (**Figure 4**). During the development of hollowed root, 4’-deoxyflavone and chrysin contents, and the expression of enzymes related to 4’-deoxyflavone biosynthesis increased together with MeJA content. The pith parenchymal cells were dead in stage 2 roots. The 4’-deoxyflavone contents and the expression of enzymes related to 4’-deoxyflavone biosynthesis remained higher compared with those in stage 1 roots. These results were consistent with the development of heartwood in trees, which also have a transition zone. Parenchymal cells in the transition zone exhibit a temporary spike in secondary metabolic activity and accumulate flavonoids (Xiao et al., 2020).

The accumulating pattern of pinocembrin chalone and pinocembrin was different from that of the 4’-deoxyflavones and chrysin (**Figure 4**). Pinocembrin chalone and pinocembrin are intermediate metabolites of 4’-deoxyflavone biosynthesis, and pinocembrin chalone and pinocembrin contents increased significantly during stage 2, then slowly decreased during stages 3 and 4. Synthesis of the pinocembrin chalone and pinocembrin enzymes showed the same expression pattern. SbCHS and SbCHI were significantly induced during stage 2, and were maintained during stages 3 and 4. Pinocembrin dehydrogenates to Chrysin,and SbFNSII-2 catalyzes dehydrogenation of pinocembrin to synthesize chrysin. The accumulation and expression pattern of chrysin and SbFNSII-2 were consistent with 4’-deoxyflavone and their enzymes, indicating that SbFNSII-2 is key regulator of the accumulation of 4’-deoxyflavones during the development of hollowed root.

JAs can be induced by senescence (Ghorbel et al., 2021). Dead parenchymatous cells were discovered arranged in rows in stage 2 roots. MeJA content was induced compared with 2-year-old roots. These results indicate that senescence of xylem cells might be induced by JAs. JAs regulate downstream gene expression and flavonoid accumulation, leading to the formation of phellem tissue and the development of hollowed root.

### Accumulation of MeJA and 4’-deoxyflavones may be regulated by senescence and pathogens

Significant increases were observed in the MeJA and 4’-deoxyflavone contents as well as the expression of enzymes related to 4’-deoxyflavone biosynthesis between stage 3 and stage 4 roots, indicating that the accumulation of MeJA and 4’-deoxyflavones is induced in several ways. In stage 3 roots, dead xylem was present but no phellem tissue was observed. The dead xylem was rotten in stage 4 roots, and the phellem tissue, which prevents attack by pathogens (Machado et al., 2013), formed. MeJA is induced by pathogens (Yu et al., 2019). 4’-Deoxyflavones, such as baicalein and wogonin, have anti-fungal, anti-bacterial, and antioxidant activities (Coll et al., 2014; Tian et al., 2018; Palierse et al., 2021; Zhu et al., 2021). Rotting xylem is an ideal medium for fungi and bacteria. The significant increases in MeJA and 4’-deoxyflavones in stage 4 roots suggest that the accumulation of MeJA and 4’-deoxyflavones may also be related to resistance to fungi and bacteria by *S. baicalensis.* Based on these results, we hypothesized that the accumulation of MeJA and 4’-deoxyflavones may be regulated by senescence and pathogen infection in roots with a hollow.

The RNA-seq results supported this hypothesis. Our RNA-seq results revealed 23 genes related to pathogen resistance and PCD, 10 genes related to the development of xylem and phellem tissues, and 7 genes related to 4-deoxyflavone biosynthetic enzymes. Sb01g32610 and Sb01g53940 were annotated as homologs of AtMC1 and AtMC9. AtMC1 is a key regulator of PCD in *A. thaliana* (Watanabe and Lam, 2005) that participates in PCD induced by aging and pathogens (Coll et al., 2014). AtMC9 also regulates PCD in *A. thaliana* by participating in PCD during xylem and vessel development (Bollhöner et al., 2013). Sb01g32610 and Sb01g53940 expression was induced in 3-year-old roots with a hollow (**Supplementary Table 2**). Sb01g32610 and Sb01g53940 expression was significantly induced during stage 2 than stage 1 during the development of hollowed root. The expression of Sb01g32610 and Sb01g53940 decreased during stages 3 and 4, compared with stage 3 (**Figures 6A, B**). This result indicates that the expression of Sb01g32610 and Sb01g53940 was induced by senescence of xylem. Sb08g10770 was annotated as a homolog of AtWOX4, a key regulator of the development and maintenance of vascular cambium (Zhou et al., 2015) that participates in the development of periderm in *A. thaliana* (Xiao et al., 2020). Sb08g10770 expression was induced in 3-year-old roots with a hollow (**Supplementary Table 2**). Sb08g10770 expression was induced during stage 4 of hollowed root development, indicating that ternary phellem tissue only develops during stage 4. Sb01g06320, Sb01g16540, and Sb01g16570 are three pathogen-resistance genes. Sb01g06320 was annotated as a homolog of AtPGIP2, which are polygalacturonase inhibiting proteins (PGIPs) induced by pathogen infection. PGIPs are plant proteins that counteract fungal polygalacturonases, and fungal polygalacturonases are important fungal virulence factors (Ferrari et al., 2006). AtPGIP2 expression can be induced by *Botrytis cinerea* infection and by jasmonate. Overexpression of AtPGIP2 significantly reduces *Botrytis* disease symptoms in *A. thaliana* (Ferrari et al., 2003). Sb01g16540 and Sb01g16570 were annotated as homologs of AtRPP8 and AtRPP13. These are two R genes that respond to pathogen infection in *A. thaliana*. The expression of Sb01g06320, Sb01g16540, and Sb01g16570 was highly induced during stage 4, indicating that a pathogen infection occurred during stage 4. These results are consistent with MeJA and 4’-deoxyflavone contents, which increased significantly during stage 4 than stage 3.

In summary, hollowed root of *S. baicalensis* is comprised of dead secondary xylem surrounded by ternary phellem tissue. The development of hollowed root was divided into 4 stages based on its anatomical structure. The accumulation of 4’-deoxyflavones and the induction of 4’-deoxyflavone biosynthetic enzymes occurred during stage 2, and increased significantly during stage 4 than during stage 3. Exogenous MeJA induced the accumulation of 4’-deoxyflavones in *S. baicalensis* roots. The accumulation of 4’-deoxyflavones is regulated by MeJA during development of hollowed root. Based on the RNA-seq and metabolite results, we discovered that the accumulation of JAs and 4’-deoxyflavones in *S. baicalensis* roots was related to senescence and pathogen infection, so the genes related to pathogen resistance and senescence were explored.

## Data availability statement

The data presnted in the study are deposited in the National Genomics Data Center (https://ngdc.cncb.ac.cn/?lang=en), accession number is PRJCA011917.

## Author contributions

DG, WX, LG, and LH designed the experiments. DG, HD, WL, HL, and RW performed the experiments. DG and WX wrote the manuscript. DG and MJ analyzed RNA-seq data. DG analyzed the data. All authors contributed to the article and approved the submitted version.
