# Supplementary material for: MeJA regulates the accumulation of baicalein and other 4’-hydroxyflavones during the hollowed root development in Scutellaria baicalensis
Source: Front Plant Sci. 2023 Jan 6;13:1067847. doi: 10.3389/fpls.2022.1067847 (PMC9853287; doi:10.3389/fpls.2022.1067847)
Supplement: Supplementary file 1 [file Presentation_1.pptx]

## Slide 1
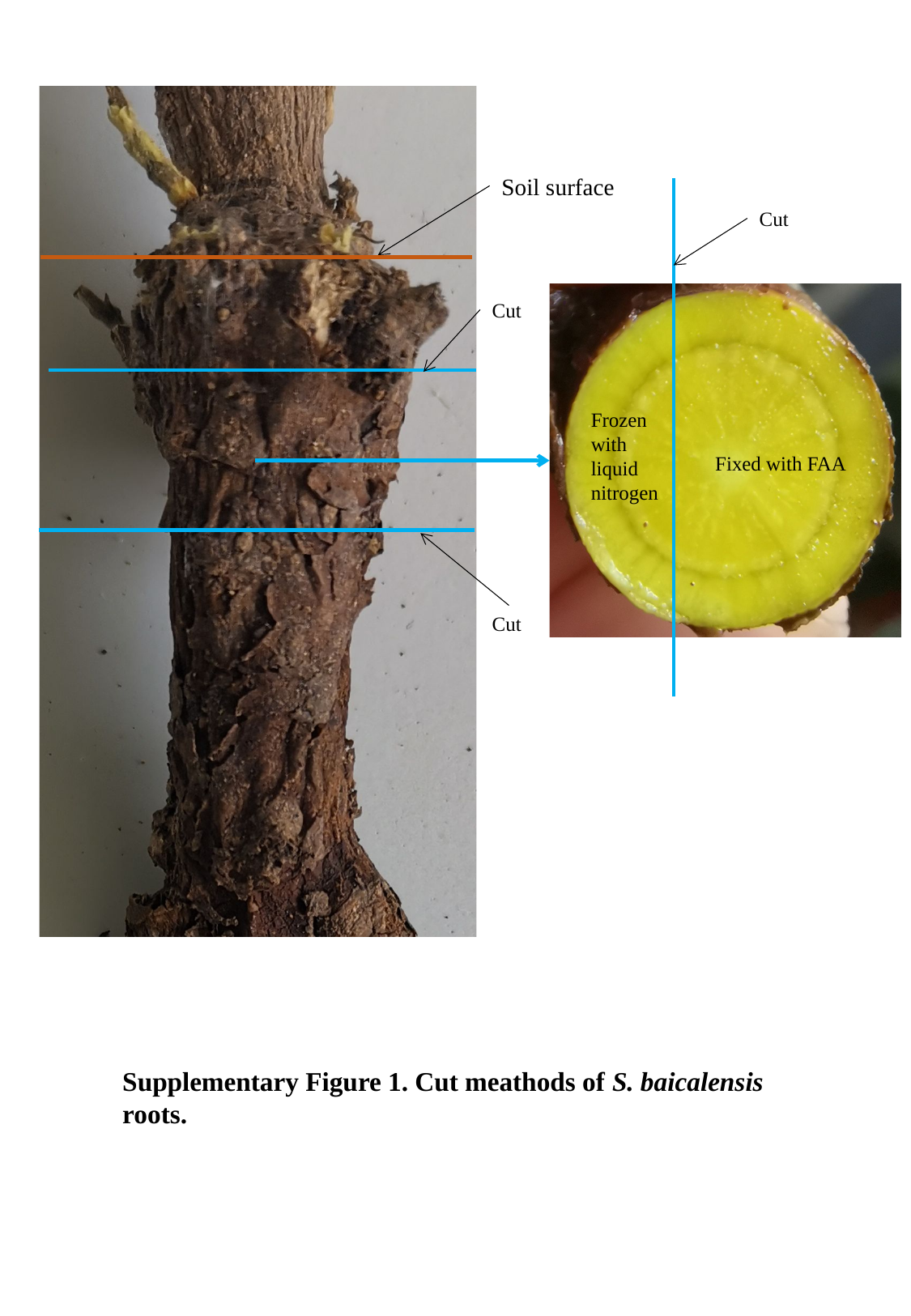

Soil surface
Cut
Cut
Frozen with liquid nitrogen
Fixed with FAA
Cut
Supplementary Figure 1. Cut meathods of S. baicalensis roots.

## Slide 2
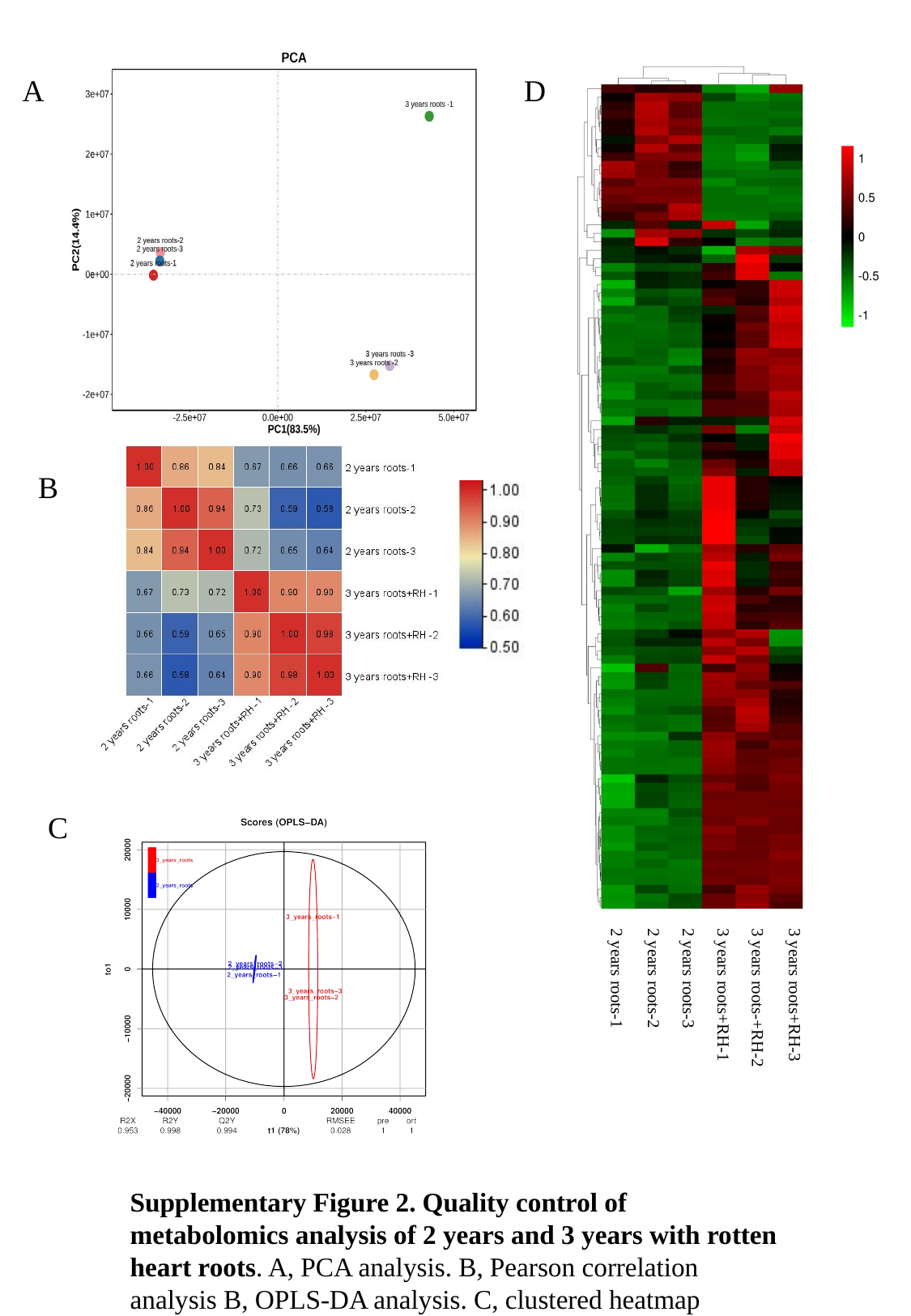

A
D
B
C
2 years roots-2
2 years roots-3
2 years roots-1
3 years roots+RH-1
3 years roots-+RH-2
3 years roots+RH-3
Supplementary Figure 2. Quality control of metabolomics analysis of 2 years and 3 years with rotten heart roots. A, PCA analysis. B, Pearson correlation analysis B, OPLS-DA analysis. C, clustered heatmap

## Slide 3
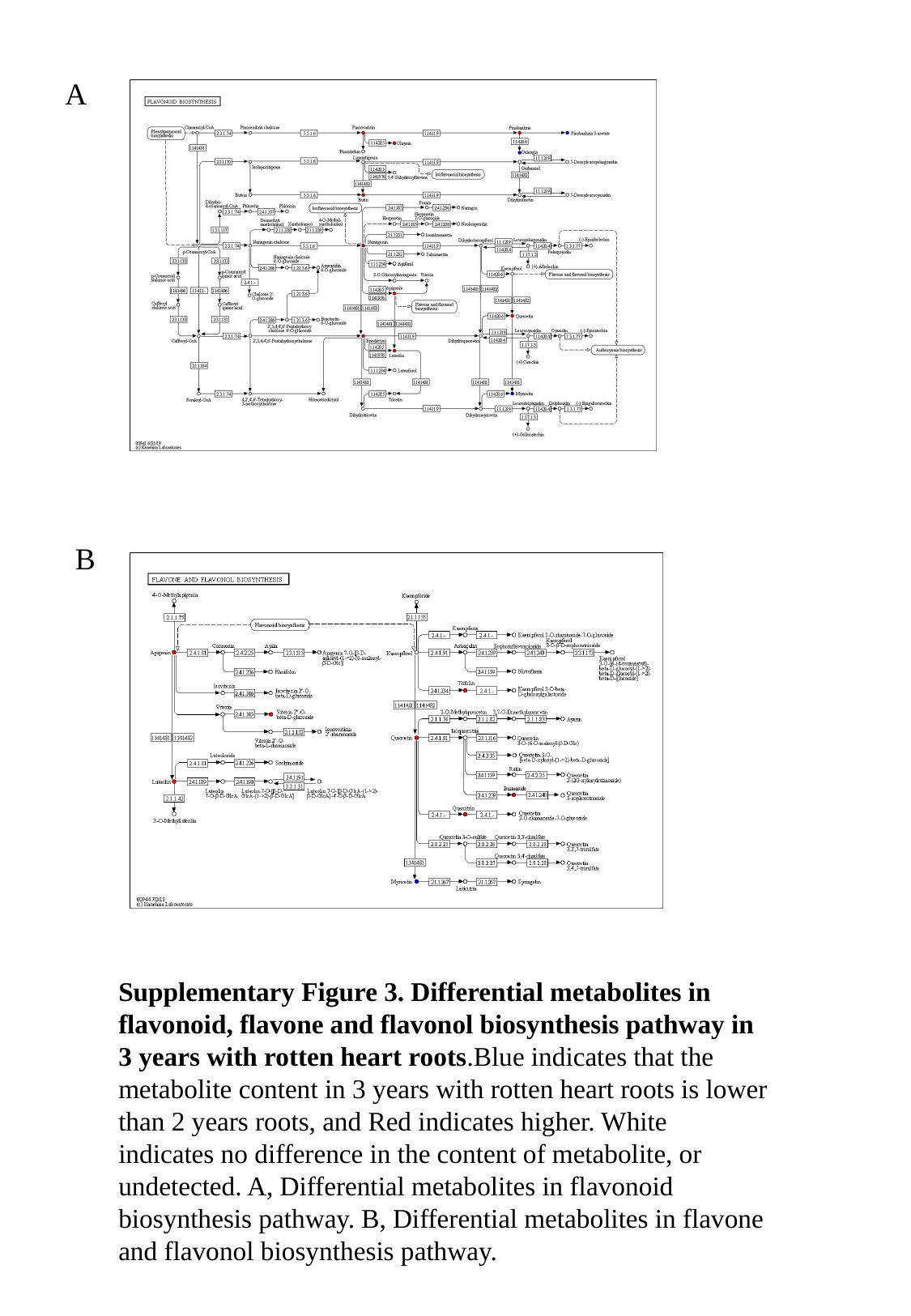

A
B
Supplementary Figure 3. Differential metabolites in flavonoid, flavone and flavonol biosynthesis pathway in 3 years with rotten heart roots.Blue indicates that the metabolite content in 3 years with rotten heart roots is lower than 2 years roots, and Red indicates higher. White indicates no difference in the content of metabolite, or undetected. A, Differential metabolites in flavonoid biosynthesis pathway. B, Differential metabolites in flavone and flavonol biosynthesis pathway.

## Slide 4
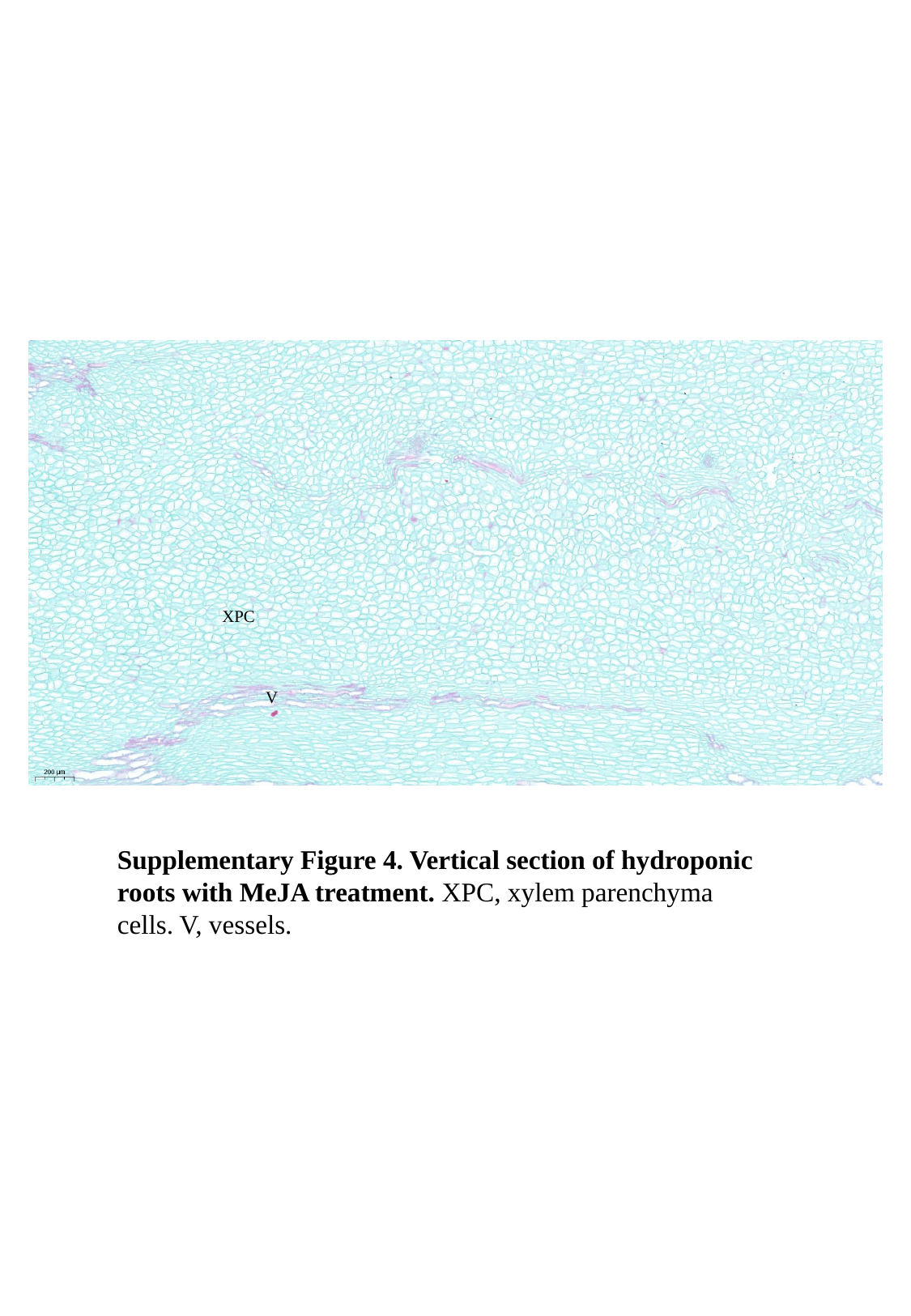

XPC
V
Supplementary Figure 4. Vertical section of hydroponic roots with MeJA treatment. XPC, xylem parenchyma cells. V, vessels.

## Slide 5
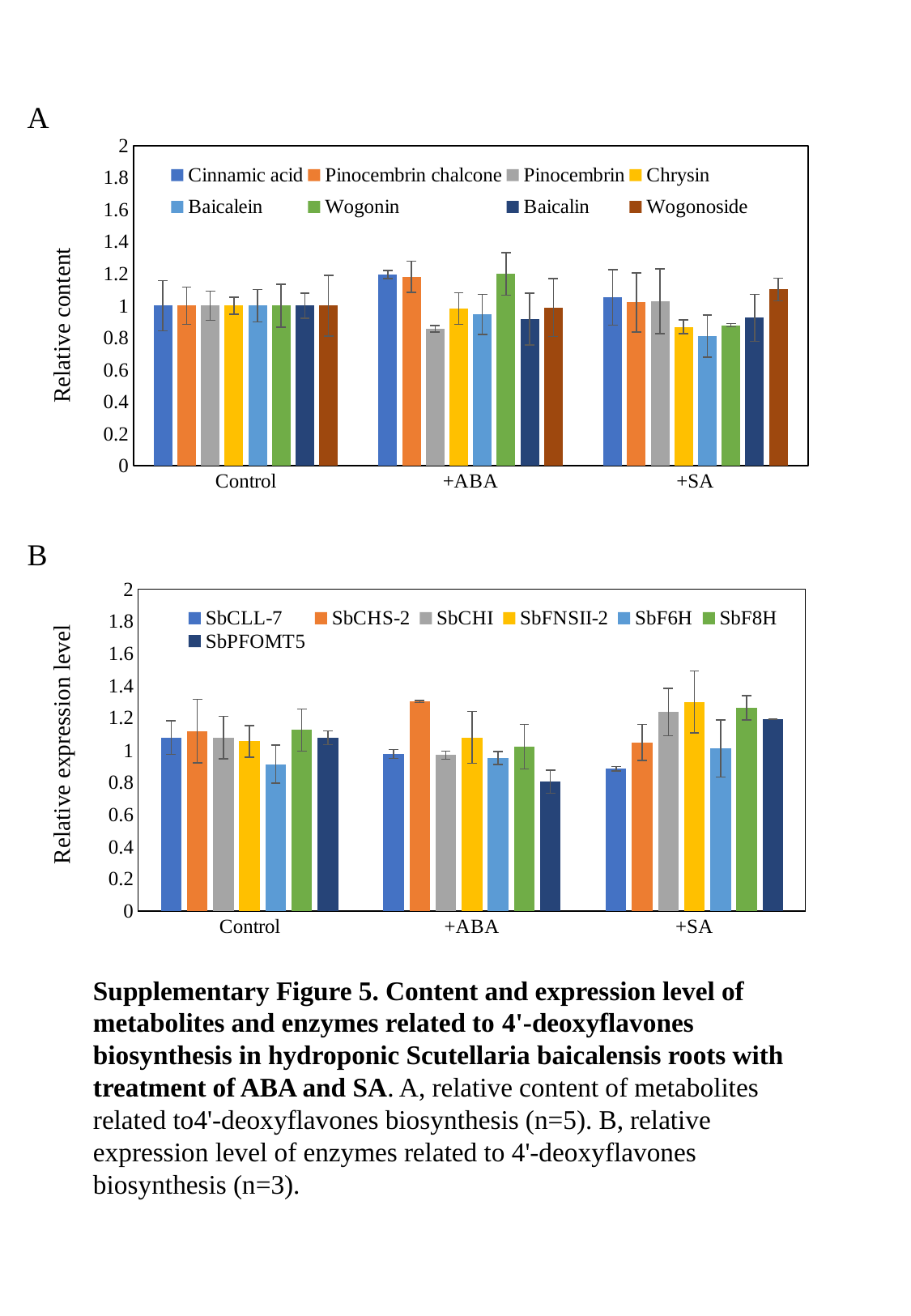

A
### Chart
| Category | Cinnamic acid | Pinocembrin chalcone | Pinocembrin | Chrysin | Baicalein | Wogonin | Baicalin | Wogonoside |
|---|---|---|---|---|---|---|---|---|
| Control | 1.0 | 1.0 | 1.0 | 1.0 | 1.0 | 1.0 | 1.0 | 1.0 |
| +ABA | 1.19467103117943 | 1.17981192858837 | 0.856278520839115 | 0.981667580824683 | 0.946059201176092 | 1.19864892029443 | 0.916230224310737 | 0.98760375245086 |
| +SA | 1.05152657488048 | 1.02046481791853 | 1.02785518100326 | 0.867569340382445 | 0.809519127076291 | 0.877985149254668 | 0.923841087302378 | 1.10119478959249 |Relative content
B
### Chart
| Category | SbCLL-7 | SbCHS-2 | SbCHI | SbFNSII-2 | SbF6H | SbF8H | SbPFOMT5 |
|---|---|---|---|---|---|---|---|
| Control | 1.0787057 | 1.11966343628179 | 1.0792427400041 | 1.05539852009958 | 0.913381678725009 | 1.12505149317255 | 1.07726472 |
| +ABA | 0.976175891164647 | 1.30262853289683 | 0.969009035810797 | 1.07951580873973 | 0.951362957270388 | 1.02162861855786 | 0.80454185619784 |
| +SA | 0.884710467049226 | 1.04835973990433 | 1.23643619625681 | 1.2992859437049 | 1.00997979501936 | 1.2626009190129 | 1.19464132769007 |Relative expression level
Supplementary Figure 5. Content and expression level of metabolites and enzymes related to 4'-deoxyflavones biosynthesis in hydroponic Scutellaria baicalensis roots with treatment of ABA and SA. A, relative content of metabolites related to4'-deoxyflavones biosynthesis (n=5). B, relative expression level of enzymes related to 4'-deoxyflavones biosynthesis (n=3).

## Slide 6
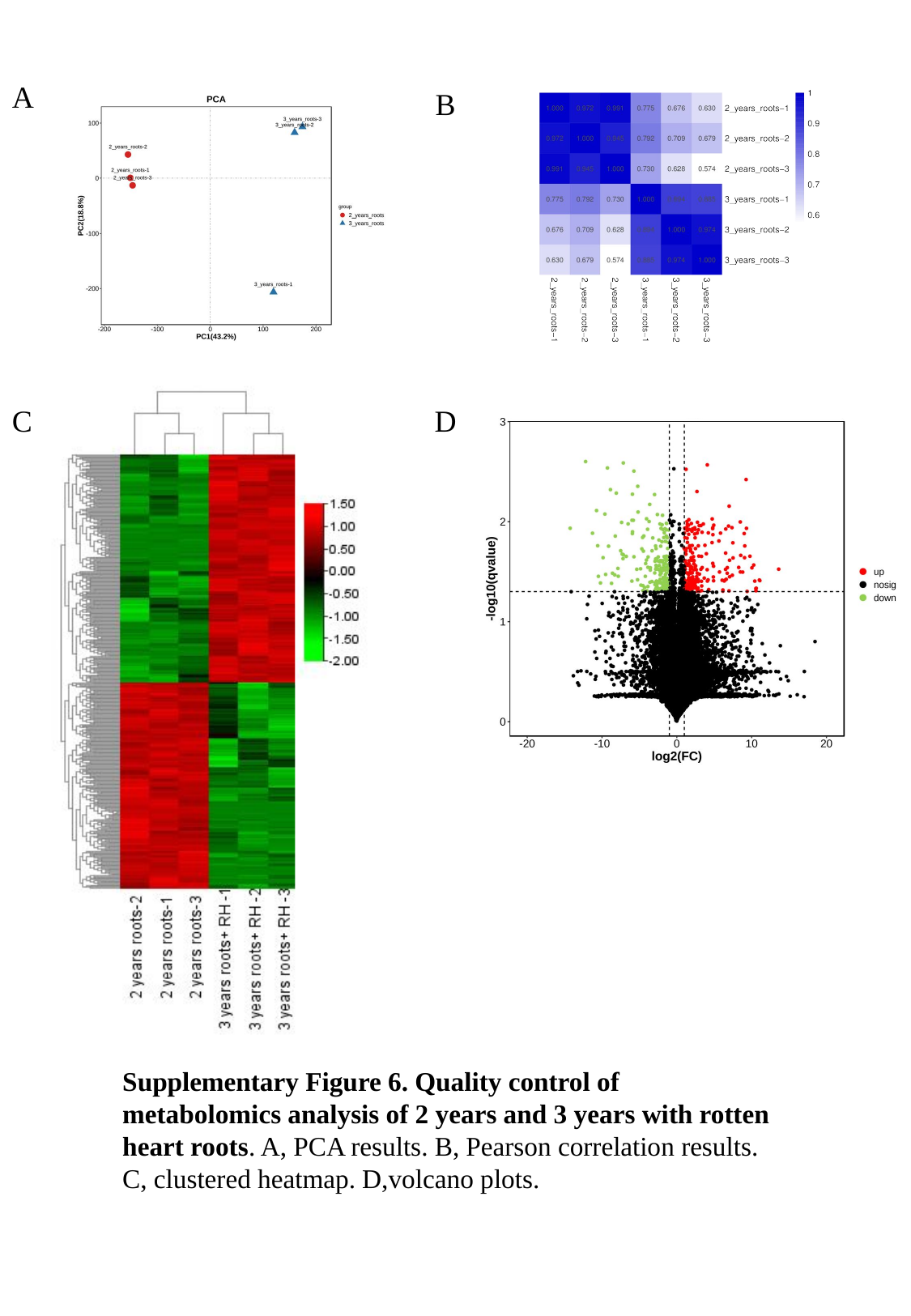

A
B
C
D
Supplementary Figure 6. Quality control of metabolomics analysis of 2 years and 3 years with rotten heart roots. A, PCA results. B, Pearson correlation results. C, clustered heatmap. D,volcano plots.
